# Blockade of muscarinic receptors impairs the retrieval of well-trained memory

**DOI:** 10.3389/fnagi.2014.00063

**Published:** 2014-04-08

**Authors:** Shogo Soma, Naofumi Suematsu, Satoshi Shimegi

**Affiliations:** ^1^Department of Health and Sport Sciences, Graduate School of Medicine, Osaka UniversityOsaka, Japan; ^2^Department of Health and Sport Sciences, Graduate School of Frontier Biosciences, Osaka UniversityOsaka, Japan

**Keywords:** acetylcholine, dicyclomine, muscarinic m1 receptor, the cholinergic hypothesis, retrieval, well-trained cognitive memory, Long-Evans rat

## Abstract

Acetylcholine (ACh) is known to play an important role in memory functions, and its deficit has been proposed to cause the cognitive decline associated with advanced age and Alzheimer's disease (the cholinergic hypothesis). Although many studies have tested the cholinergic hypothesis for recently acquired memory, only a few have investigated the role of ACh in the retrieval process of well-trained cognitive memory, which describes the memory established from repetition and daily routine. To examine this point, we trained rats to perform a two-alternative forced-choice visual detection task. Each trial was started by having the rats pull upward a central-lever, which triggered the presentation of a visual stimulus to the right or left side of the display monitor, and then pulling upward a stimulus-relevant choice-lever located on both sides. Rats learned the task within 10 days, and the task training was continued for a month. Task performance was measured with or without systemic administration of a muscarinic ACh receptor (mAChR) antagonist, scopolamine (SCOP), prior to the test. After 30 min of SCOP administration, rats stopped manipulating any lever even though they explored the lever and surrounding environment, suggesting a loss of the task-related associative memory. Three hours later, rats were recovered to complete the trial, but the rats selected the levers irrespective of the visual stimulus, suggesting they remembered a series of lever-manipulations in association with a reward, but not association between the reward and visual stimulation. Furthermore, an m1-AChR, but not nicotinic AChR antagonist caused a similar deficit in the task execution. SCOP neither interfered with locomotor activity nor drinking behavior, while it influenced anxiety. These results suggest that the activation of mAChRs at basal ACh levels is essential for the recall of well-trained cognitive memory.

## Introduction

A loss of cholinergic function in the central nervous system has been demonstrated to contribute significantly to the cognitive decline associated with advanced age and Alzheimer's disease (AD; reviewed, Bartus, 2000). This hypothetical causality is known as “the cholinergic hypothesis,” which was initially presented over 30 years ago (Bartus et al., 1982). Brains from patients of old age or AD-related cognitive dysfunction have consistently shown damage or abnormalities in cholinergic pathways that appeared to correlate well with the level of cognitive decline (Whitehouse et al., 1981, 1982). This hypothesis has since served as the basis for the majority of treatment strategies and drug development approaches for AD to date. Thus, acetylcholine (ACh) is now firmly entrenched as a key factor to prevent and cure the disease, although the etiology still remains to be less understood.

The typical symptom pattern of AD begins with memory loss, especially for recent events (short-term or working memory). With AD progression, at least one other cognitive deficit occurs, including apraxia, agnosia, or a disturbance in executive functioning. Symptoms of AD eventually include impairment of well-learned functions expressed frequently and almost automatically in daily living. For example, apraxia is defined as a higher-order motor disturbance of goal-directed behavior characterized by an inability to perform previously learned movements in the absence of weakness or sensory defects (Edwards et al., 1991). Agnosia is defined as an inability to recognize objects that are familiar or well-known (Bennett, 2000; Abe et al., 2007).

Previous studies testing the cholinergic hypothesis have mostly focused on functions relevant to recently acquired memory (Zarrindast et al., 1996; Marti Barros et al., 2004; Soares et al., 2006; Piri and Zarrindast, 2011). Those studies demonstrated that subclasses of ACh receptors (AChRs) involved in memory retrieval depended on the memory type. For example, pre-test administration of a muscarinic AChR (mAChR) antagonist impaired the retrieval of memory acquired from contextual fear conditioning (Soares et al., 2006). On the other hand, nicotinic AChRs (nAChRs) contributed to memory retrieval in the inhibitory avoidance task (Zarrindast et al., 1996; Marti Barros et al., 2004; Piri and Zarrindast, 2011). To date, however, it still remains unclear about the relationship between the cholinergic system and memory established by daily routine. The purpose of this study was to examine whether the cholinergic hypothesis explains deficits of well-learned memory retrieval.

To examine the role of ACh in well-learned memory functions, we applied a two-alternative forced-choice (2AFC) visual detection task to rats as a test of memory function, because this task requires multiply-associative learning. Here, rats were required to memorize the association between lever-manipulation and a reward. This associative relationship was additionally associated with a visual stimulus. This design mimics instrumental activity of daily living. For example, we raise a coffee cup to make coffee and then to drink, where raising the cup gets increasingly associated with drinking indirectly and directly with each repetition. Thereby, just when we wanted to drink a cup of coffee, the memories structured centering on the cup are efficiently retrieved in a goal-directed manner on the basis of external information. Thus, the 2AFC visual detection task is suitable for constructing the multiply-associative memory structure established by our daily routine. After completion of the task learning within 10 days and a subsequent task training period for one month, the effects of scopolamine (SCOP), an mAChR antagonist, on task performance was examined. We define the memory used in daily routine after completion of the learning as a well-trained memory.

SCOP was chosen because of comprehensive comparison of ours with a lot of previous studies examining the effects on memory-related functions other than well-trained memory and various cognitive functions (Hodges et al., 2009; Klinkenberg and Blokland, 2010). We also tested the effects of blood-brain barrier (BBB) impermeable scopolamine methyl-bromide (methyl-SCOP) and an m1-specific AChR antagonist to exclude the several possible effects of SCOP such as salivation and attention (Klinkenberg and Blokland, 2011). We adopted systemic administration of SCOP to mimic AD patients, as they generally suffer from diffuse and global impairment of cholinergic functions (Whitehouse et al., 1981, 1982).

SCOP caused severe impairment of performance, as rats temporarily and specifically lost task-related memories about the two associations, but recalled them over time in the order they were learned. Our results demonstrate that activation of mAChRs with basal levels of ACh is essential for the retrieval of well-trained cognitive memory established by daily routine.

## Materials and methods

### Animals

Twenty male Long-Evans rats (Institute for Animal Reproduction, Ibaraki, Japan) were kept on 12 h light/dark cycles, and tests were performed during the light cycle. All experimental protocols were approved by the Research Ethics Committee of Osaka University, and all procedures were carried out in compliance with the policies and regulations of the guidelines approved by the Animal Care Committee of the Osaka University Medical School and National Institutes of Health guidelines for the care of experimental animals.

### Water control

Animals had *ad libitum* access to water during weekends, but obtained water only by performing the task correctly on other days. Signs of possible dehydration were monitored (reduced skin tension, sunken eyes, and marked variations in general behavior), but none were observed in all animals. To ensure adequate hydration, we weighed each animal at the beginning and end of each experimental session and compared the weight to a standard weight updated weekly. If the weight measured after the session was <90% the standard weight, the animal would be temporarily taken out of the study and given *ad libitum* access to water until the weight recovered. This condition never occurred. Average ± *SD* of body weights were 296 ± 14 g and 431 ± 12 g before and after learning and training phases, respectively.

### Apparatus in 2AFC task

**Figure 1:**
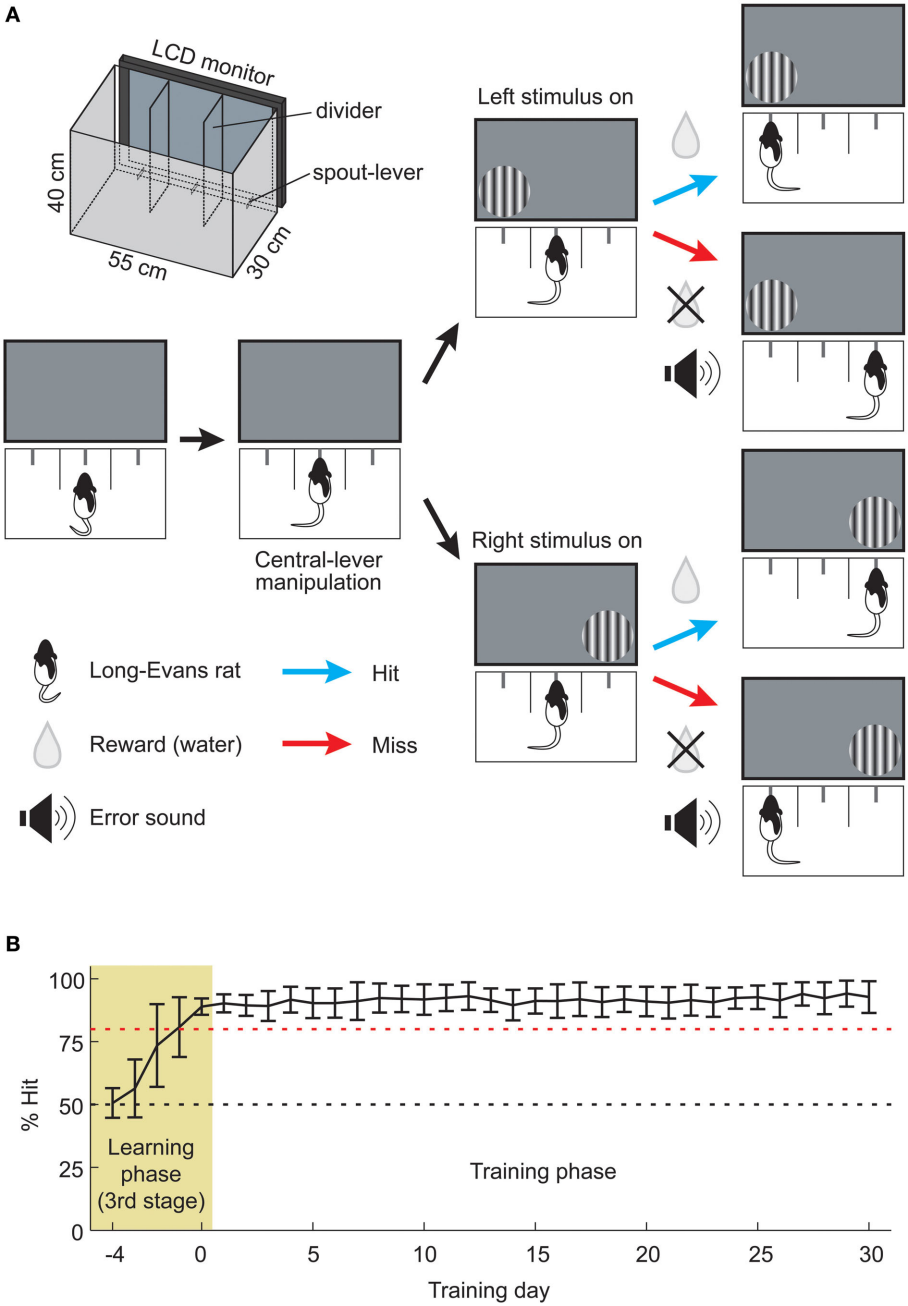
**Assessment of well-trained memory by the 2AFC visual detection task. (A)** Schema of the visual stimulus detection task based on a 2AFC paradigm. First, the rat pulled central-lever up for presenting the visual stimulus. Next rat was required to detect the stimulus and pull the corresponding choice-lever upward. When the animal made a correct choice (Hit), water was rewarded from the choice-lever. In the case of an incorrect choice (Miss), only an error signal was given. The schematic illustration of the choice-box is depicted in upper left (see Methods for details). **(B)** Animal's learning and training processes. Animals (*n* = 20) learned a visual stimulus detection task over 5 learning days (pale yellow area, Learning phase), and the same task was subsequently continued for another 30 days (open area, Training phase). Red dashed line indicates 80% Hit. Data are presented as mean ± *SD*.

The choice-box (Figure 1A; 30 cm long × 40 cm high × 55 cm wide) was made as described in detail elsewhere (Soma et al., 2013c). The front side of the box was translucent and faced an LCD monitor (mean luminance, 30 cd/m^2^). The box was divided by translucent walls to produce three connected areas that each had a spout-lever in its center: a central-lever in the middle area and choice-levers in the other two. To shorten the learning period, we used the spout-lever as an operandum (Kimura et al., 2012) by which rats were able to learn rapidly that pulling it upward correlated with acquisition of the reward. The reward volume was changed by controlling the open time of a solenoid valve that was manipulated by a PC. Speakers attached to the monitor gave signals indicating task initiation and auditory feedback of a task error. Animal behavior was monitored through a webcam. Software for the experimental control and stimulus presentation was written in MATLAB (Mathworks, Natick, MA, USA) with extensions from the Psychophysics Toolbox (Brainard, 1997; Pelli, 1997).

### Behavioral task for the memory retrieval test

**Figure 2:**
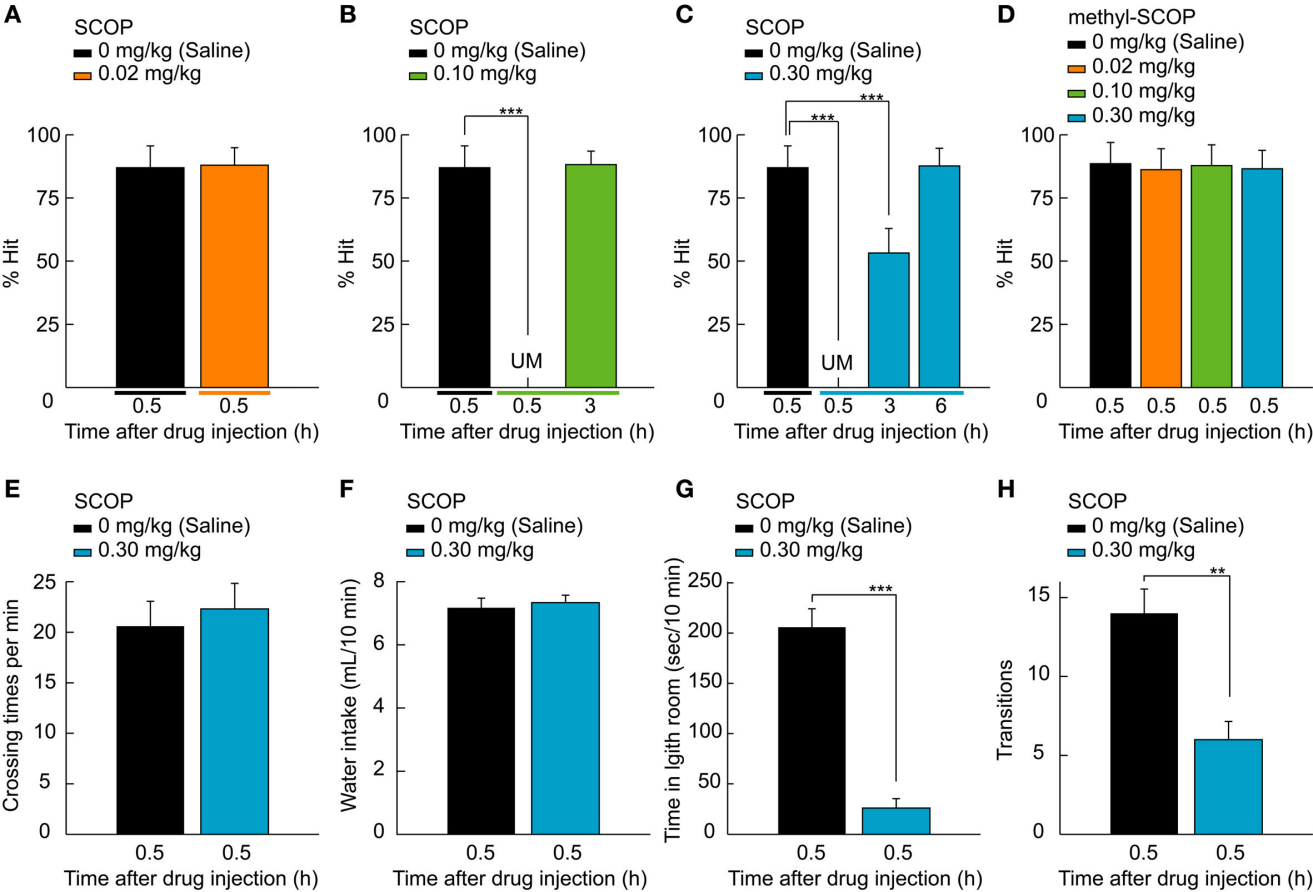
**SCOP-induced temporary blackout of well-trained memory. (A–D)** Rat performance tested after the injection of saline and various doses of SCOP or methyl-SCOP (0.02, 0.1, and 0.3 mg/kg). Rats never manipulated the lever 0.5 h after 0.10 or 0.30 mg/kg SCOP administration, as behavioral performance was unmeasurable **(**UM; **B,C)**. In contrast, 0.02 mg/kg SCOP and all doses of methyl-SCOP did not affect performance **(A,D)**. At 3 h after SCOP injection, rats remembered the task paradigm at 0.10 mg/kg and above **(B,C)**, but the association between the lever and reward only at 0.30 mg/kg **(C)**. **(E,F)** High concentration of SCOP (0.30 mg/kg) affects neither the amount of locomotor activity level measured in an open field nor the water intake from a regular water bottle. **(G,H)** SCOP injection at 0.30 mg/kg influenced anxiety. Data are presented as mean + *SD*; ^**^P < 0.01. ^***^P < 0.001. *n* = 10 rats/group.

**Figure 3:**
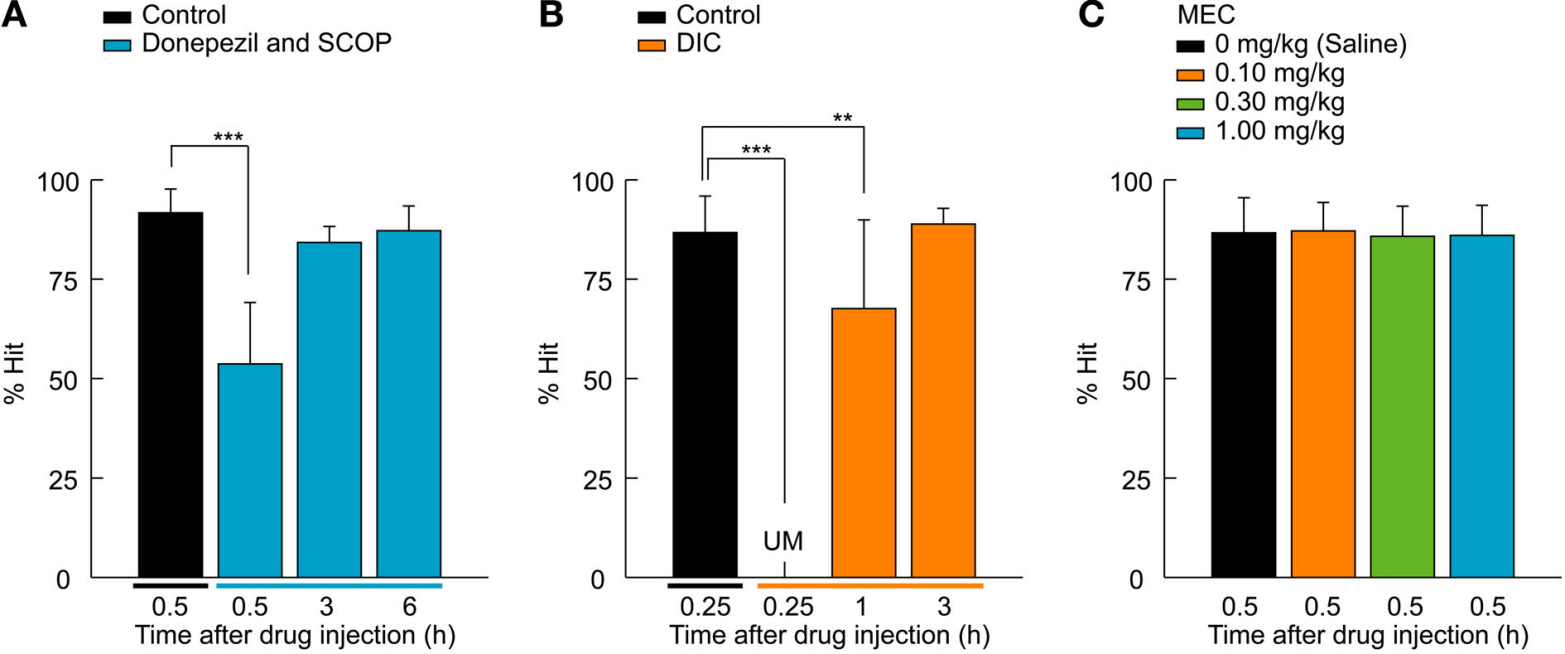
**M1 AChR-mediated retrieval function of the 2AFC task. (A)** A cholinesterase inhibitor, donepezil (1.0 mg/kg), was systemically administered to rats a half-hour before SCOP injection (0.30 mg/kg). Donepezil attenuated the effect of SCOP on the performance of the 2AFC task, as rats were able to recall the association between lever-manipulation and reward even 0.5 h after the SCOP injection and perform the 2AFC task after 3 h equally to that of the control condition. *n* = 5 rats/group. **(B)** DIC (8.0 mg/kg), an m1-specific AChR antagonist, was tested using the 2AFC task. The same effects as SCOP were seen, but in a shorter time course. *n* = 5 rats/group. **(C)** MEC, an nAChR antagonist, did not influence rat performance. *n* = 10 rats/group. Data are presented as mean + *SD*; ^**^P < 0.01. ^***^P < 0.001.

We used the 2AFC visual detection task in order to examine the cholinergic effect on memory retrieval. Rats began each trial by pulling upward the central-lever, which triggered the presentation of a circular patch of static sinusoidal grating to the right or left side of the display monitor (Figure 1A). The viewing distance of the stimulus center from the central-lever was 13 cm. The parameters of vertical grating (spatial frequency, 0.1 cycles/degree; diameter, 70 degrees) were determined according to our previous study (Soma et al., 2013c). The stimulus presentation continued until the rat pulled upward any choice-lever. The grating location was randomly changed from trial to trial. Upon a correct choice, rats received a reward of 2–5 μ L of water. Upon an incorrect choice, rats only received an audible sound (200–500 Hz). All animals performed at least 100 trials for 20 min under the no drug condition (median, 181 trials; range, 100–300 trials). Rat performance (% Hit) was calculated every 5 min by dividing the number of correct trials by that of total trials. In the recovery experiments (Figures 2B,C, 3A,B), rats were tested for the task performance at 0.5, 3, and 6 h after drug injection, in which, each test were conducted for 20 min. In the intervals between the test, rats were returned to their home cages.

### Learning and long-term task training periods

Training procedures for learning the 2AFC visual detection task are described in detail elsewhere (Soma et al., 2013c). Briefly, rats were trained in three stages. In the first stage (1–2 days), all rats (*n* = 20) learned to obtain fluid delivered from the spout-lever by pulling it upward in a single choice-box isolated from the other areas. A rat was put into the choice-box 1 h per day, in which the bigger reward (10 μ L) was delivered automatically every 30 s or by pulling the spout-lever up. The rat was easily able to learn how to acquire the reward by heuristic manipulation of the spout-lever. In the second stage (3–6 days), the rats learned how to initiate and obtain fluid during the trial in the normal choice-box used for the 2AFC task (see Figure 1A). The rat learned to pull the central-lever up before the choice-lever (task initiation), which presented a grating. When either choice-lever was pulled up before the central-lever, an error-sound (200–500 Hz) was given as an instructive feedback signal. After the task initiation, the grating presentation continued until the rat pulled upward the correct choice-lever by which fluid was delivered. Thereby, a rat learned sequential manipulation of the central and choice-levers to obtain the reward. In the third stage (6–10 days), rats learned that the fluid supply is associated with the visual stimulus. During the training period of the task, rats learned the following in a stepwise fashion: association between (1) lever-manipulation and the reward, and (2) visual stimulus and the reward. The task device was developed to efficiently obtain well-trained memory by securing a sufficient length of training period and number of task repetitions (Soma et al., 2013c). Furthermore, our device provided a great number of repetitive training within a short time without task interruption by human handling.

We judged the completion of task learning when the success rate reached more than 80% in the total trials of one day. This stage is indicated by the pale yellow area in Figure 1B (Learning phase, days −4 to 0; Third stage, 6–10 days). The task was continued for another 30 days in the training phase (open area, days 1–30; Figure 1B) for the purpose of examining the effects of the cholinergic blockade on well-learned behavioral functions established daily routine. The task conditions of the training phase were the same as those of the memory retrieval test, and rats performed 300 trials within a session (60-min cutoff).

### Pharmacological treatments

The role of the cholinergic system in the retrieval function was assessed using ACh-relevant drugs. All drugs were administered intraperitoneally with an injection volume of 1 ml/kg prior to the behavioral test. Drugs were dissolved in 0.9% saline to create appropriate stock solutions. SCOP (Sigma-Aldrich, St. Louis, MO, USA), an antagonist of mAChRs that permeates the BBB, and methyl-SCOP (Sigma-Aldrich, St. Louis, MO, USA), an antagonist of mAChRs that is impermeable of the BBB, were given at three doses (0.02, 0.10, and 0.30 mg/kg) and administered 0.5 h prior to the test. The doses of SCOP were chosen based on previous studies (Chopin and Briley, 1992; Kaneko and Thompson, 1997; Hodges et al., 2009; Klinkenberg and Blokland, 2011). In the rescue study, donepezil (Eisai, Ibaraki, Japan), a cholinesterase inhibitor, was given at 1.0 mg/kg (Takahata et al., 2005; Wise et al., 2007). At 0.5 h after the donepezil administration, 0.30 mg/kg SCOP was given 0.5 h prior to the 2AFC test. Other pharmacological studies were carried out using 8.0 mg/kg dicyclomine (DIC; Sigma-Aldrich, St. Louis, MO, USA), an m1-specific AChR antagonist (Bartko et al., 2011), and 0.1, 0.3, and 1.0 mg/kg mecamylamine (MEC; Sigma-Aldrich, St. Louis, MO, USA), an nAChR antagonist. The former and latter were given 0.25 and 0.5 h before the test, respectively. In all pharmacological studies, 0.9% saline was injected as a control at the same time points as the drug administration.

All pharmacological studies were conducted in within-subjects design. Rats were assigned to either SCOP/methyl-SCOP test group (Figures 2A–D) or other drugs test group (Figure 3). In the SCOP/methyl-SCOP test, data were obtained from 10 rats. After the 30-day training phase, the rats were tested by a crossed Latin square design including 2 control conditions (1: vs. SCOP and 2: vs. methyl-SCOP), 3 doses of SCOP conditions and 3 doses of methyl-SCOP conditions (total 8 conditions/rat). Individual rats were tested for all the 8 conditions in the pseudo-random order through days 31–38. These ten rats were subsequently subjected to the other types of tests including open field test, water intake, and light-dark room test by a crossed Latin square design (Figures 2E–H). This battery of tests consisted of 6 conditions, derived from 2 drug conditions (control and 0.3 mg/kg SCOP) for 3 test types (2 × 3 conditions/rat). The all the testes were done at 0.5 h after the administration of saline or SCOP through days 39–44, in which the order of the tests were pseudo-randomly assigned to individual rats.

Among 10 rats of other drugs test group, 5 rats were assigned to the donepezil test (Figure 3A), another 5 to the DIC test (Figure 3B), and the both of them (*n* = 10) were used in the MEC test (Figure 3C). These rats too were tested by a crossed Latin square design including 2 control conditions (1: vs. donepezil + SCOP, or vs. DIC; 2: vs. MEC), either the donepezil or DIC condition, and MEC conditions at 3 doses in the pseudo-random order through days 31–36 (total 6 conditions/rat). In all the pharmacological tests, each rat underwent one test a day.

### Open field test

The locomotor activity level was measured in an open field. The test field was a circle of 60 cm in diameter and was surrounded by a wall of 30 cm in height. The entire field was monitored from above by a webcam which was connected to a PC for the recording of behavior. Each rat was placed in the center of the test field, and the rat's behavior was recorded in video. The activity level was analyzed off-line by an observer blind to the experimental conditions. The circular test field was divided into nine zones; each rat's activity level was measured as the total number of crossovers between zones (i.e., crossing a boundary line between zones with two hind paws) for 5 min. The open field test was conducted under two drug conditions, saline and SCOP (0.30 mg/kg).

### Measurement of water intake

The degree of dehydration was examined by measuring the water intake for 5 min. At 0.5 h after the administration of saline or 0.30 mg/kg SCOP, rats were placed in the home cage and had *ad libitum* access to water from a regular bottle. Water intake was measured as the weight loss of the bottle.

### Light-dark room test

A light-dark room test was performed to examine whether SCOP influences anxiety and impairs light perception. The test was conducted under two drug conditions, saline and SCOP (0.30 mg/kg). The apparatus was a plastic box (48 cm long × 24 cm high × 27 cm wide) consisting of two equal-size compartments in which rats could freely move between through an open gate (10 cm high × 10 cm wide). The sides of the box were opaque, and the top was covered with an opaque lid for the dark room condition and a transparent lid for the light room condition. The light room was illuminated with a 27-W fluorescent lamp set 40 cm above the box (mean luminance, 40 cd/m^2^). Rats were placed in the center of the light room and allowed to freely explore the box. After the rats entered the dark room, the number of transitions and the time spent in the light room were measured for 10 min.

### Statistical analyses

We used the Kolmogorov-Smirnov test to test the goodness of fit of a given set of data to a theoretical distribution. In the 2AFC task, normally distributed data were analyzed by one-way analysis of variance (ANOVA) for repeated measures followed by *post hoc* Dunnett's test with comparison to the control group. Data not showing a normal distribution were analyzed by the non-parametric Friedman test. Further statistical analyses for individual groups were carried out by the Wilcoxon signed-rank test with the Bonferroni correction. Statistical analyses of other measures obtained from the open field test, the light-dark room test, and water intake were carried out using the Wilcoxon signed-rank test.

## Results

### Rats acquire well-trained cognitive memory by repetitive training over the long-term

In order to investigate the functional roles of ACh in the retrieval process of well-trained memory established from daily routine, we adopted operant conditioning based on a 2AFC visual detection task that requires cognitive procedures in a behavioral choice-box (Figure 1A; Movie S1; Soma et al., 2013c). The improvement of learning was assessed as the success rate (% Hit), and the learning completion was defined as % Hit larger than 80% (pale yellow area, Learning phase; Figure 1B). The training phase was set to last one month after completion of the learning task. During this time, the rats continued to perform the visual stimulus detection task at a high performance (open area, Training phase; Figure 1B). After the 30-day training phase, the performance of the well-trained rats was tested with or without SCOP (0.02, 0.10, and 0.30 mg/kg; Figure 2A).

### Scop impairs the retrieval of well-trained memory

SCOP had a concentration-dependent effect on the retrieval function (Figures 2A–C). Low concentration of SOCP (0.02 mg/kg) did not affect behavioral performance (*P* = 0.406, Wilcoxon signed-rank test; Figure 2A), whereas middle and high concentrations (0.10 and 0.30 mg/kg) had a strong negative effect on performance (χ^2^ = 60.2, *df* = 2, *P* < 0.001, Figure 2B; χ^2 = 109.1^, *df* = 3, *P* < 0.001, Friedman test; Figure 2C). We were not able to measure rat performance 0.5 h after SCOP administration, because the rats never manipulated the lever at the time (*P* < 0.001, Figure 2B; *P* < 0.001, Wilcoxon signed-rank test with Bonferroni correction; Figure 2C), suggesting that they had forgotten what they had learned, i.e. the association between the lever-manipulation and task initiation/reward acquisition (see Movie S2). However, SCOP has been known to dilate pupil which might impair visual perception. To confirm whether SCOP caused its effects by acting on the central nervous system, we tested methyl-SCOP, which is an mAChR antagonist that is impermeable to the BBB. The same doses of methyl-SCOP as SCOP (0.02, 0.10, and 0.30 mg/kg) did not affect behavioral performance [*F*_(3, 77)_ = 0.4, *P* = 0.766, one-way ANOVA for repeated measures; Figure 2D]. To rule out the possibility that SCOP influences motor function and dehydration, we performed an open field test and a measurement of water intake, respectively. SCOP (0.30 mg/kg) neither affected the amount of locomotor activity (*P* = 0.313, Wilcoxon signed-rank test; Figure 2E) nor water intake (*P* = 0.732, Wilcoxon signed-rank test; Figure 2F). These results suggest that the unmeasurable performance of the 2AFC task was induced via mAChRs in the central nervous system and not due to functional changes in the peripheral nervous system or motor impairment. SCOP has been known to increase anxiety. Therefore, we conducted the light-dark room test. The SCOP-injected group stayed significantly longer in the dark room than the control group (time in light room, *P* < 0.001; transitions, *P* < 0.01, Wilcoxon signed-rank test; Figures 2G,H), indicating an anxious tendency.

Recovery from the SCOP-induced task impairment showed different time courses in a dose-dependent manner. At 3 h after the drug administration, the task performance was completely recovered (*P* = 0.429, Wilcoxon signed-rank test with Bonferroni correction; Figure 2B) at 0.10 mg/kg. On the other hand, the performance at 0.30 mg/kg was chance level (*P* < 0.001, Wilcoxon signed-rank test with Bonferroni correction; Figure 2C), because rats were able to pull the central-lever upward to start the task, but selected the choice-lever randomly (Movie S3). This result argues that under this situation rats could remember the association between the central-lever manipulation and task initiation, but not the association between the visual stimulus and reward. A further 3 h later (6 h after the drug administration), rats could perform the task at control level (*P* = 0.315, Wilcoxon signed-rank test with Bonferroni correction; Figure 2C). Thus, recall of the association memories did not occur simultaneously, suggesting that the accessibility of the memories differ, with lower accessibility requiring more ACh for memory retrieval.

### Rescue of temporal amnesia by donepezil

Next we investigated if a cholinesterase inhibitor, donepezil, was able to rescue the impairment of the task performance (Figure 3A). Rats were systemically injected with donepezil (1.0 mg/kg) 0.5 h before the 0.30 mg/kg SCOP administration. The 2AFC visual detection task was conducted using the same time course as the SOCP alone injection experiments described in Figure 2C. SCOP still exerted an effect [*F*_(3, 77)_ = 83.7, *P* < 0.001, one-way ANOVA for repeated measures; Figure 3A], but the effect was attenuated by the pre-administration of donepezil. % Hit under the co-administration condition was chance level (*P* < 0.001, Dunnett's *post hoc* test) at 0.5 h after the SCOP injection, whereas it was unmeasurable in the SCOP-only condition (see Figure 2C). Additionally, the recovery time of the behavioral performance was shortened, as 3 h after the drug co-administration, rats could perform the visual stimulus detection task without significant differences from the control condition (3 h, *P* = 0.411; 6 h, *P* = 0.486; Dunnett's *post hoc* test; Figure 3A).

### M1-AChR-mediated temporal amnesia

To investigate whether m1-AChRs, an mAChR subtype, contribute to the retrieval function, we tested the effect of DIC (8.0 mg/kg), an m1-specific AChR antagonist (Figure 3B). DIC caused the same effect as SCOP (χ^2^ = 44.9, *df* = 2, *P* < 0.001, Friedman test; Figure 3B), but over a shorter time course. Rats suffered a temporary blackout with regards to the association between the lever and reward 0.25 h after the DIC administration (*P* < 0.001, Wilcoxon signed-rank test with Bonferroni correction), but remembered the association at 1 h after the administration (*P* < 0.01, Wilcoxon signed-rank test with Bonferroni correction). After 3 h, the behavioral performance was completely recovered (*P* = 0.353, Wilcoxon signed-rank test with Bonferroni correction). These observations suggest that the memory retrieval function of ACh is at least mediated by m1-AChRs. Finally, we examined whether nAChRs are involved in the retrieval function by using MEC, an nAChR antagonist. All tested doses (0.1, 0.3, and 1.0 mg/kg) of MEC had no effect on rat performance [*F*_(3, 77)_ = 0.6, *P* = 0.634, one-way ANOVA for repeated measures; Figure 3C].

## Discussion

To investigate the possibility that cholinergic deficiency is involved in various cognitive deficits of progressed AD patients, that is, impairment of well-learned functions expressed frequently in daily living, we examined the effects of cholinergic blockade on well-trained memory established by daily routine. After rats learned the 2AFC visual detection task within 10 days, the task training was continued every day for a month. After the training phase, the effect of systemic administration of SCOP on the 2AFC visual detection test was examined. The results are summarized as follows: (1) middle and high doses of SCOP (0.1 and 0.3 mg/kg) caused an impairment of task performance, as rats showed no task initiation due to no lever-manipulation at 0.5 h and only haphazard selection of the choice-lever after task initiation at 3 h post SCOP administration; (2) rats started to perform the task over time in the order the tasks were learned; (3) pre-administration of donepezil attenuated the SCOP-induced temporal task impairment; and (4) blockade of m1-AChRs but not nAChRs caused the impairments similar to those from SCOP administration.

### Role of ACh in memory retrieval

Although many researchers have examined how ACh contributes to memory functions such as encoding and consolidation (Miranda and Bermudez-Rattoni, 1999; Nail-Boucherie et al., 2000; Wallenstein and Vago, 2001; Gold, 2003; Rogers and Kesner, 2003; Bang and Brown, 2009; Pang et al., 2010; Pepeu and Giovannini, 2010), there are few studies that have investigated the role of the cholinergic system in memory retrieval (Zarrindast et al., 1996; Marti Barros et al., 2004; Soares et al., 2006; Piri and Zarrindast, 2011). Since those studies focused mainly on aversively motivated tasks, it remains poorly understood how other type of memory is modulated by ACh and which type of AChR subtype mediates the action. This study provides the first evidence that activation of mAChRs, at least m1-AChRs, is necessary to retrieve well-trained memory. M1-AChRs-dependent memory retrieval has also been reported for recently acquired memory formed during aversively motivated tasks. Soares et al. (2006) demonstrated that pre-test administration of DIC impaired the retrieval of memory acquired from contextual fear conditioning but not from inhibitory avoidance task, suggesting task-dependent differential contributions of m1-AChRs. On the other hand, the memory retrieval in inhibitory avoidance task seems to be mediated via nAChRs, because the latency of mice for entering a dark room that contained footshock is increased by the systemic administration of nicotine (Zarrindast et al., 1996; Marti Barros et al., 2004; Piri and Zarrindast, 2011) and decreased by the antagonistic blockade of nAChR (Zarrindast et al., 1996; Marti Barros et al., 2004). Thus, retrieval functions can be mediated by different AChRs depending on the type of memory.

### Memory formed by a long-term daily training

In general, animals trained more than the number of the training session required for the completion of task learning is considered as overtrained animals. Although the overtrained animals reach the plateau in the task performance, overtraining-induced plastic changes in nervous system have been known to continue and vary in quantity and quality depending on the number of session (Luft and Buitrago, 2005), for example, the overtraining of water maze increased synaptogenesis of hippocampal mossy fibers according to the session number (Ramírez-Amaya et al., 1999). Thus, neuronal plastic changes are thought to continue in contrast with an impression of the term “overtraining” that training-induced plastic change plateaus not followed by additional change. Moreover, it has been known that extensive training causes a transformation from goal (reward)-directed actions into habitual responses of animals (Yin and Knowlton, 2006). For example, hungry rats are trained to press a lever in order to obtain sucrose pellets, and post training devaluation of the pellets by conditioned taste aversion causes a reduction in lever-pressing after moderate training but not after extensive training (Dickinson, 1985), suggesting that the behavior is still goal-directed after the moderate training and becomes habitual after the extensive training. In that case, brain areas, networks, and synaptic transmission responsible for task-relevant memories are thought to change depending on the training conditions such as the number and period of training. Therefore, the cholinergic sensitivity of the task-relevant memory should be evaluated in the same task at different amounts of the training. In the present study, rats seemed to perform the task in a reward-directed manner since rats stopped executing the task when rat's thirst was quenched with the water even after 30 days training phase. However, there are important questions about whether the reward-directed behavior is transformed into habitual responses if the training period increased further, and if so, whether cholinergic blockade is effective on the task performance. Those points should be examined in further study.

### Side effects of scop

SCOP has been reported to have cognitive as well as non-cognitive effects (Bushnell et al., 1997; Sipos et al., 1999; Mirza and Stolerman, 2000; Phillips et al., 2000; Hodges et al., 2009; Klinkenberg and Blokland, 2010). In the present study, we performed an open field test, and a measurement of water intake in order to rule out the possibility that SCOP influences motor function, and dehydration, respectively. SCOP (0.3 mg/kg) did not impair motor function, as the amount of locomotor activity in the open field was not different between control and SCOP conditions (Figure 2E). In the choice-box, SCOP-administered rats seemed to have reduced activity (Movie S2) in comparison with control rats (Movie S1). However, this observation does not mean that SCOP caused the low activity, because the same behavioral pattern and activity were observed when naïve rats unlearning the task were put in the choice-box. We propose that the speedy action and high activity of well-trained rats are more likely due to their reward-directed and well-motivated behavior.

It is known that SCOP has a “dry mouth” side effect that reduces salivation (Dai et al., 1991; Shiraishi and Takayanagi, 1993; Hodges et al., 2009). However, this effect is unlikely to explain the absence of task initiation seen in the present report for several reasons. First, the reward was water, not dry food; second, the water intake was not affected by administration of 0.3 mg/kg SCOP (Figure 2F); and third, methyl-SCOP, which has the same peripheral side effects as SCOP (Hodges et al., 2009), did not impair execution of the task (Figure 2D).

Since visual information processing is modulated by ACh (Goard and Dan, 2009; Kang et al., 2013; Soma et al., 2013a,b,c), the light-dark room test was conducted to examine whether SCOP impairs the light perception. If SCOP did induce visual impairment, rats should stay in the dark and light rooms equally. However, rats stayed in the dark room longer, suggesting that SCOP-administered rats were able to perceive light (Figures 2G,H). In addition, Tsui and Dringenberg (2013) found that SCOP at a dose of 1.0 mg/kg, which is higher than that used in the present study, did not impair performance in visual discrimination tasks that used striped textures as stimuli.

The other side effect of SCOP is pupil dilatation, which might influence the task performance by making rats blind in bright light. However, the pupil dilatation does not seem to be responsible for SCOP-induced deficit in the task execution because methyl-SCOP was reported to dilate the pupil twice as effective as SCOP (Jones and Higgins, 1995) but not impaired the task performance in this study (Figure 2D). Therefore, SCOP seems not to impair the visual detection in our experimental conditions.

In the light-dark room test, SCOP-injected rats stayed significantly longer in the dark room compared to control rats, implying the appearance of anxiogenic-like behavior (Klinkenberg and Blokland, 2010). A similar symptom has been reported in previous rat studies observing rats (Smythe et al., 1996; Hughes et al., 2004) as well as humans (Curran et al., 1991), showing increased anxiety as well as amnestic effects by SCOP. However, anxiogenic-like behavior was not a major cause of our observations, because SCOP-injected rats moved around and explored the surrounding environment inside the choice-box including the levers (Movie S2).

### Possible influence of scop on cognitive functions

SCOP is known to impair cognitive functions such as working memory and attention (Bushnell et al., 1997; Hodges et al., 2009). In the present study, working memory was not required to perform the 2AFC visual detection task, but attention might be needed depending on the stimulus condition. In a visual detection task, the difficulty of the stimulus detection is correlated with demand of attentional capacity. For example, SCOP slightly impaired the performance in an easy visual cue detection task without distracter but severely disrupted it when distracter was simultaneously presented (Jones and Higgins, 1995). Since task difficulty can be manipulated by changing the stimulus parameters (Soma et al., 2013c), we set the easiest stimulus condition (see Materials and Methods) to lessen the possibility that SCOP effects on attention contributed to the task performance. The cholinergic system is also involved in sustained attention (Bushnell et al., 1997; Parikh et al., 2007). In those studies, animals were required to sustain their attention to detect an unexpected visual cue, under which cue detectability was associated with cortical ACh content (Parikh et al., 2007). However, in our study, the sustained attention for stimulus detection is not required, because the visual stimulus was presented by the rat's central-lever manipulation. Klinkenberg and Blokland (2011) reported that m1-specific AChR antagonist is better than SCOP to cause mnemonic deficits because of the small effect on attentional function. Since DIC as well as SCOP caused severe impairment of the task performance in this study, we believe that the SCOP-induced deficit in the task execution is not due to an impairment of the working memory and attentional functions.

### Neural mechanisms underlying cholinergic regulation of memory retrieval

How is ACh involved in the retrieval of well-trained memory? Various kinds of long-term memory including personal experience (Penfield, 1952) and experimentally-experienced visual stimuli (Hasegawa et al., 1998) are stored in the neocortex and retrieved by glutamatergic top-down signals from the prefrontal cortex (Hasegawa et al., 1998). These corticocortical feedback connections, which convey top-down signals, project to extragranular layers where mAChRs are richly expressed (Levey et al., 1991). Consistent with this structure, we found that SCOP, but not MEC, impaired the recall of well-trained memory. Cortical ACh originating in the nucleus basalis of the basal forebrain (Saper, 1984) could allow focused memory to be easily retrieved by the glutamatergic system by regulating the dynamics of the neuronal network via activation of mAChRs but not nAChRs.

### Significance of the present study

One of the most critical problems in patients with AD is their forgetting important information that should be well-known (agnosia), such as the name of children and spouses (Bennett, 2000) or well-somatized (apraxia). The donepezil treatment has been known to slightly reduce the frequency of memory-retrieval deficits in family relationships (Abe et al., 2007) suggests that ACh is necessary for recalling well-trained memory expressed frequently and almost automatically in daily living, and that the deficit of cholinergic functions leads to the above cognitive dysfunctions. On the other hand, cholinesterase inhibitors have been known to be less effective on late phase of AD patients, suggesting that cholinergic neurons are mostly depleted at that time. Therefore, our experimental protocol seems to imitate well the symptoms of late AD patients, and moreover can induce it reversibly. We believe that the reversibly-induced late AD animal model is useful for new drug screening and investigation of the neuronal mechanisms underlying amnesia. Thus, the present results deepen our understanding of the roles of ACh in memory functions and give clinical implications on diseases associated with ACh deficiency.

## Author contributions

Shogo Soma and Satoshi Shimegi designed the research, Shogo Soma and Naofumi Suematsu collected the data and performed the analyses, and Shogo Soma and Satoshi Shimegi wrote the paper.

### Conflict of interest statement

The authors declare that the research was conducted in the absence of any commercial or financial relationships that could be construed as a potential conflict of interest.

## Supplementary material

Movie S1 **Rat performance of the 2AFC visual detection task under control condition**. When a rat pulled the central-lever up, a grating patch appears on the right or left side of the display monitor. The animal was required to detect the stimulus and pull the corresponding choice-lever to obtain the reward through the lever.

Movie S2 **Impairment of the retrieval of well-trained memory by scopolamine**. The rat never manipulated the lever 0.5 h after administration of scopolamine at high concentrations (0.30 mg/kg).

Movie S3 **Recovery from the scopolamine-induced task impairment**. At 3 h after 0.30 mg/kg scopolamine administration, the rat set out to manipulate the lever but selected the choice-lever randomly, resulting in chance level of the task performance (see Figure 2C).
